# Using an exploratory sequential mixed methods design to adapt an Illness Perception Questionnaire for African Americans with diabetes: the mixed data integration process

**DOI:** 10.1080/21642850.2021.1976650

**Published:** 2021-09-13

**Authors:** Olayinka O. Shiyanbola, Deepika Rao, Daniel Bolt, Carolyn Brown, Mengqi Zhang, Earlise Ward

**Affiliations:** a Division of Social and Administrative Sciences, School of Pharmacy, University of Wisconsin–Madison, Madison, WI, USA; b Department of Educational Psychology, University of Wisconsin–Madison, Madison, WI, USA; c Department of Health Outcomes and Pharmacy Practice, University of Texas at Austin, Austin, TX, USA; d School of Pharmacy, University of Wisconsin–Madison, Madison, WI, USA; e School of Nursing, University of Wisconsin–Madison, Madison, WI, USA

**Keywords:** Illness perceptions, African Americans, mixed methods, diabetes, data integration

## Abstract

**Background:**

Although qualitative methods have been used to develop quantitative behavioral health measurements, studies rarely report on the exact development process of these questionnaires. In this methodological paper, we highlight the procedure of a mixed data integration process in using qualitative data to create quantitative questionnaire items.

**Methods:**

We used an exploratory sequential mixed methods study design to culturally adapt the Illness Perception Questionnaire-Revised (IPQ-R) and address the sociocultural contexts of African Americans with type 2 diabetes. Forty African Americans with type 2 diabetes taking oral diabetes medication completed the qualitative focus groups and 170 participants completed the quantitative phase (surveys). Using the ‘building approach’ to integration, qualitative themes from the focus groups were matched to survey domains based on the self-regulatory model. Qualitative themes assessing perceptions of diabetes among African Americans were used to develop new survey items for a culturally adapted IPQ-R, as well as adapt original survey items.

**Results:**

Important themes included the effect on friend/family relationships, lifestyle changes, food experiences (consequences domain), importance of medications (treatment control), comparisons with family members (illness coherence), fear, future worries, and anger (emotional representations). A new domain, ‘sociocultural influences’ was added to the adapted questionnaire based on qualitative themes of race and racism on provider roles, personal control, and community influences. Merging and integration of the qualitative and quantitative phases, (reported via a joint display) showed evidence of congruence between the illness perceptions from the qualitative focus groups and scores on the survey items.

**Conclusion:**

The use of mixed methods allowed for the development of a robust and patient-centered questionnaire. Future research should consider psychometric testing of the adapted IPQ-R, so that it may be used in addressing illness perceptions among African Americans.

## Introduction

Diabetes is one of the leading causes of death in the United States. Compared to non-Hispanic whites, African Americans are twice as likely to have been diagnosed with diabetes and experience higher burden of diabetes-related complications, leading to increased morbidity and mortality (Spanakis & Golden, 2013). Optimal glycemic control and clinical outcomes among patients with type 2 diabetes are commonly associated with long-term use of therapeutic drugs and medication adherence (Aloudah et al., 2018). The rate of medication adherence is 25% lower among African Americans with diabetes, compared to non-Hispanic whites after standardizing factors, such as insurance coverage, access to the care, and quality of care (García-Pérez, Alvarez, Dilla, Gil-Guillén, & Orozco-Beltrán, 2013; Patel et al., 2016).

One significant patient factor that affects medication adherence and diabetes self-management is illness and medication beliefs. Based on the Extended Self-Regulatory Model (ESRM), patients’ illness perceptions influence self-management behaviors and in turn affect outcomes (Phillips, Leventhal, & Leventhal, 2011). The ESRM (also called the Common Sense Model) is a theoretical framework useful in understanding the influence of illness perceptions on self-management behaviors. Studies have reported a higher medication adherence when healthcare providers discuss and address illness and medication beliefs with patients, subsequently leading to better disease self-management and improved health outcomes (Phillips et al., 2011; Sweileh et al., 2014). Importantly, recent studies provide evidence that illness perceptions play a significant role in self-management behaviors and outcomes among African Americans with type 2 diabetes (Broadbent, Donkin, & Stroh, 2011; Rovner & Casten, 2018; Skelly et al., 2006).

To improve self-management behaviors and clinical outcomes among African Americans, it is important for health care providers to understand how their cultural contexts and beliefs might influence their perceptions about diabetes and shape their self-management behaviors. Several studies have used a validated tool, Illness Perception Questionnaire-Revised (IPQ-R) (Abubakari et al., 2012; Arat et al., 2016; Ward, Wiltshire, Detry, & Brown, 2013), to identify and assess the different components of diabetes beliefs. Although this tool has been widely used in various patient populations, it has not been used to assess beliefs among African Americans with diabetes, and does not account for the underlying sociocultural factors that might influence the illness beliefs of African Americans. The IPQ-R developed in a European population is sometimes ineffective in accurately assessing diabetes beliefs among African Americans because their unique perceptions and culturally influencing factors are not addressed adequately (Abubakari et al., 2012). A study evaluating the psychometric properties of the IPQ-R among African-origin populations discussed the need for modifying several items to better capture the cultural influences of the population (Abubakari et al., 2012). To our knowledge, no study has developed a culturally adapted illness perception questionnaire to characterize African Americans’ illness beliefs.

## Methods

Mixed methods research involves qualitative and quantitative data collection and analysis approaches within the same study (Creswell & Clark, 2017). Moreover, these research approaches involve integration of the qualitative and quantitative results to provide an enhanced and comprehensive answer to a research question. Data integration, a process of systematically merging quantitative and qualitative data can occur in numerous ways and at various levels during the study design, methods (data collection, data analysis), and reporting/data interpretation stages (Creswell & Clark, 2017). An exploratory sequential design is a mixed methods study design, where the quantitative phase of data collection and analysis follows the qualitative phase of data collection and analysis (Fetters, Curry, & Creswell, 2013).

Mixed method approaches offer opportunities to study contextual factors such as culture, perceptions, beliefs qualitatively and develop quantitative measures. Although mixed method approaches have been used to develop and adapt questionnaires, the process of data integration has not been adequately described. This leads to gaps in methodological validity and a lack of connection between the qualitative and quantitative phases. Separate reporting of qualitative and quantitative data, even in the same paper does not allow the reader to gain a full understanding of the process. Moreover, data integration increases the credibility of qualitative findings if the quantitative results are congruent with the latter in an exploratory sequential design (Onwuegbuzie, Bustamante, & Nelson, 2010). Mixed method studies specifically focusing on assessing African Americans’ culturally influenced illness perceptions with detailed descriptions of the integration phase are needed. Therefore, the purpose of this study is to culturally adapt the IPQ-R to address the sociocultural contexts of African Americans with type 2 diabetes and evaluate the congruence between the qualitative and quantitative data in the mixed methods approach.

### Study design

As described above, we used an exploratory sequential mixed methods design and systematically integrated the qualitative and quantitative findings. Of the various integration approaches at the methods level, the ‘building’ approach involves using the data from one phase to inform the data collection approach of the second phase. Data integration at the reporting level includes using the ‘merging’ approach to form a ‘joint display’ (Fetters et al., 2013). A joint display is defined as, ‘bringing the quantitative and qualitative findings together through a visual means, to draw out new insights beyond the information gained from the separate quantitative and qualitative results’ (Guetterman, Fetters, & Creswell, 2015). We used the building approach to systematically develop quantitative items based on qualitative data and the merging approach to evaluate congruence between the findings of the two phases. The merging of both phases is demonstrated via the joint display.

Mixed methods are a useful tool for developing quantitative instruments as described by Onwuegbuzie et al. In this context, there are ten phases of the ‘instrument development and construct validation process’ (Onwuegbuzie et al., 2010). The first four phases of this process involve using mixed methods to conceptualize the construct of interest, identifying, and describing behaviors underlying the construct, developing the initial instrument, and pilot testing the instrument. Utilizing mixed methods in various steps of the instrument development process increases instrument fidelity by assessing appropriateness with both qualitative and quantitative data (Onwuegbuzie et al., 2010). Thus, the exploratory sequential mixed methods study design was needed in this study for increasing the validity and reliability of the adapted instrument.

### Sampling and recruitment

There were two different samples used in the study. For the qualitative phase, a purposive sample of 40 African American men and women, 45–60 years old, with a diagnosis of type 2 diabetes at least one-year prior, and who took at least one prescription diabetes medication. Participants were recruited from churches, apartment complexes and senior centers and were provided a $50 incentive. To ensure good integration between the qualitative and quantitative phase, the exclusion and inclusion criteria for the quantitative sample were the same as the qualitative sample. A convenience sample of 170 AA men and women in a Midwestern state, 45–60 years old, with a self-reported diagnosis of type 2 diabetes at least one-year prior and who took at least one prescription diabetes medication was used for the surveys. Survey participants were recruited from food pantries, churches, diabetes support groups, and clinics. Participants received $25 for completion of the survey. The study was approved by the Institutional Review Board at the researchers’ university. Informed consent was obtained from all participants.

### Qualitative data collection and analysis

**Figure 1.**
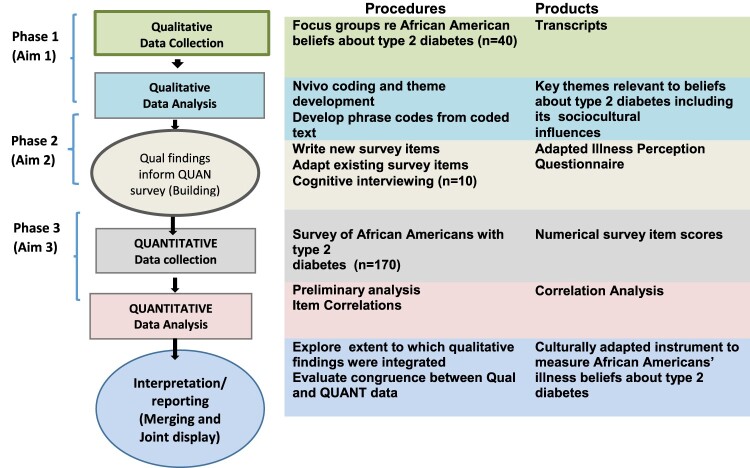
The Exploratory Sequential Mixed Methods Process for the Instrument Development of the Culturally Adapted Illness Perception Questionnaire for African Americans with Type 2 Diabetes.

The ESRM theoretically informed the study design and guided the data collection, analysis, and interpretation. The study was conducted in three phases to highlight the data stages, i.e. the qualitative, integration, and quantitative phases. The mixed methods process of data collection and analyses with the sequential steps involved is described in Figure 1. The initial qualitative phase has been described in detail in previous publications (Shiyanbola, Brown, & Ward, 2018a, 2018b, 2018c). Since research on illness perceptions of African Americans with diabetes in accordance with the ESRM was limited, the purpose of the qualitative phase was to explore their unique illness perceptions and identify the sociocultural factors that might influence these perceptions. The qualitative phase involved six 90-minute focus groups with the sample. Using phenomenology as the qualitative approach, the discussion guide was developed based on the ESRM domains (part of the focus group was deductively structured), as well as other open-ended questions to elicit other unique perceptions and topics beyond the ESRM domains. The principal investigator (PhD trained individual with extensive experience conducting focus groups) facilitated the focus groups. Focus groups were audio-recorded, transcribed professionally, analyzed by two researchers skilled in qualitative methods. Deductive content analysis was conducted on the transcripts to identify and categorize the resulting themes into ESRM domains, followed by an inductive approach using open coding to identify any new categories and themes based on other open ended questions raised (Shiyanbola, Ward, & Brown, 2018c). Analysis continued until data saturation when no new dimensions were identified in the data. Member-checking (using 4 study participants) was used to verify credibility of the findings. To do this, a summary of the findings was sent to the participants and they were asked to verify the accuracy of the findings. Participants agreed with the summary and had no changes or additions to report.

### Mixed method data integration (Building Approach)

In this exploratory sequential mixed methods design, we used the building approach for our method-level data integration, where the themes and participant quotes from the initial qualitative phase were used to write new survey items and to adapt existing survey items from the IPQ-R (quantitative phase). The latter also involved rewriting some of the existing survey items, allowing for cultural adaptation. Forty-four survey items, including new and adapted items were compiled using this approach. These items were assessed for face and content validity by an expert review from three PhD trained researchers with expertise in psychology, illness and treatment perceptions among African Americans, qualitative research, and psychometric analysis. In adapting the instrument, 29 of the 44 items were tested for further content validity through a cognitive interviewing process previously described (Shiyanbola, Bolt, Tarfa, Brown, & Ward, 2019). Five items were found to be problematic and revised accordingly. Finally, the 44 items were added to the existing 39-item IPQ-R to form the 83-item culturally adapted IPQ-R questionnaire. The adapted items followed the scoring structure of the original IPQ-R items which included a 5-point Likert scale of ‘Strongly agree’ to ‘Strongly disagree’ for all items except items under the ‘Causes’ domain. These items were scored on a binomial scale of ‘yes’ or ‘no,’ similar to the IPQ-R items under the ‘Causes’ domain.

### Quantitative data collection and analysis

Pilot testing of the newly developed items involved administering a survey that included demographic information along with the culturally adapted IPQ-R items and original IPQ-R items. The face-to-face 20 min survey was administered to the quantitative sample of 170 participants. Subsequently, data analysis included descriptive statistics, item mean scores, and item-total correlations. All statistical analyses were carried out in SPSS 26 (IBM, Amtrak).

### Reporting of mixed methods data integration (Merging Approach)

**Figure 2.**
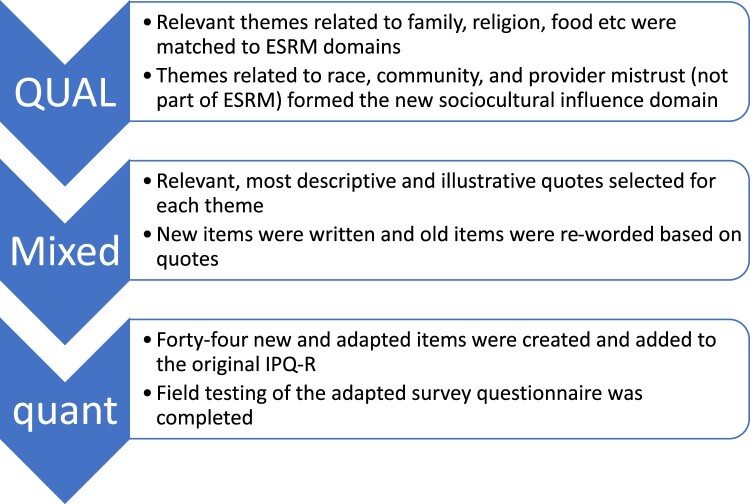
Mixed method data integration process.

Finally, data integration (merging approach) occurred at the reporting level to create a joint display of qualitative and quantitative data. This process initially required matching the qualitative themes to their corresponding IPQ-R survey item domains because of the mainly deductive approach used to conduct the content analysis of the focus group transcripts. The new themes identified by the inductive approach allowed for the formation of a new sociocultural survey domain. Under each relevant theme, sample quotes that had been used to create the specific culturally adapted items were added to the joint display, along with their corresponding items, showing the integration of the qualitative phase with the quantitative phase. After a preliminary quantitative analysis, mean item scores and item-total correlations were added to the final joint display to evaluate congruence between the two phases. The process of mixed method data integration and reporting is described in Figure 2.

### Ethics

The study was approved by the Health Sciences Institutional Review Board at the University of Wisconsin–Madison (2016-0909).

## Results

### Background characteristics of study participants

The forty participants in the focus group had a mean age of 53 years old (±4.9) and were female (61.4%). About 43.6% reported fair health. The sociodemographic and clinical characteristics of the survey sample are reported in Table 1. The sample consisted of participants who were an average of 56 years old (±7.20) and 50% had a high school education or less. Nearly 49% reported less than good health.

**Table 1.** Survey participant demographics and clinical characteristics (*n*=170).

Variable	Frequency (Percentage)	Mean (Standard Deviation)
Age		55.68 (7.20)
Sex [Female]	100 (58.8%)	
Relationship Status		
Single, never married	78 (45.9%)	
Married/ Legally recognized partnership	36 (21.2%)	
Divorced or separated	33 (19.4%)	
Other	20 (11.8%)	
Highest Level of Education		
Less than high school	36 (21.7%)	
High school graduate or GED	47 (27.6%)	
Some College or Technical School	47 (27.7%)	
College Graduate	22 (12.9%)	
Graduate Degree	14 (8.3%)	
Number of oral medications		1.87 (1.17)
Perceived Health Status		
Excellent	6 (3.5%)	
Very good	16 (9.4%)	
Good	59 (34.7%)	
Fair	71 (41.8%)	
Poor	13 (7.6%)	

### Qualitative themes

Since the qualitative results have been published previously (Shiyanbola et al., 2018a, 2018b, 2018c), this paper mainly focuses on the results of the integration process and only summarizes the qualitative findings. Themes from the focus group included contrasting ‘timeline’ perceptions of ‘diabetes not going away’ and ‘diabetes goes away if you lose weight.’ ‘Consequences’ of diabetes included changes in relationships with friends/family, lifestyle, identity, and other sociocultural factors. Two themes were matched to ‘personal control’ domain: internal and external control. Other themes included the importance of medications (treatment control domain) and understanding diabetes by family members (illness coherence domain). These themes were influenced by sociocultural factors, hence, enhancing the cultural adaptation of the questionnaire. Many themes were matched to emotional representations such as fear, anger, future doubts, depression, and acceptance of diabetes. The inductive analysis resulted in themes of race, racism and its role on providers and personal control of diabetes, race and poverty, and African American community roles. The influence of these sociocultural factors which informed both the inductive and deductive themes, lead to the cultural adaptation of the questionnaire.

### Mixed method results

The joint display showing the IPQ-R domains, qualitative themes and sample quotes, corresponding new culturally adapted items, mean item scores, and item-total correlations are presented in Table 2. The initial matching of qualitative themes to survey domains included the timeline, consequences, personal control, treatment control, illness coherence, and emotional representations domains. No culturally adapted IPQ-R item was developed for the timeline-cyclical domain as no matching themes were found in the qualitative phase. Hence, the original survey items in that specific domain were retained. According to the scoring guidelines for IPQ-R, the items under the ‘causes’ domain are not to be used as part of the scale. Instead, they are to be used as individual items and grouping variables or as separate subscales to identify perceived causes of illnesses. Therefore, although some qualitative themes related to perceived causes and corresponding culturally IPQ-R items were developed and matched to the ‘causes’ domain, they are not included here. Also, the unique themes from the inductive content analysis were combined to form the new sociocultural influences domain.

**Table 2.** Joint Display of Illness Perception Questionnaire-Revised (IPQ-R) and Culturally Adapted IPQ-R items with sample qualitative phrase codes, corresponding themes, and quantitative mean of survey items.

Illness Perception Questionnaire (IPQ-R) Survey Items^b^ and domains	Themes from Qualitative Focus Groups	Sample phrase codes	Corresponding Adapted Survey Items	Item Scores Mean (SD) (Percent agreement with item) (*n* = 170)	Item – total correlation Pearson's Correlation Coefficient
Timeline (acute/chronic): IP1^a^ My diabetes will last a short time IP2 My diabetes is likely to be permanent rather than temporary IP3 My diabetes will last for a long time IP4^a^ This diabetes will pass quickly IP5 I expect to have this diabetes for the rest of my life IP18^a^ My diabetes will improve in time	Diabetes will last a while and does not go away.	‘And I know there's no cure for being a diabetic, you’re going to always take this medication’ [Pt 20] ‘I still believe that, and I’m probably wrong, but I think once the damage is done now, I think it's no reversing it.’ {Man 3} ‘it don't really bother me no more, because there's nothing I can do with it to make it go away. The only one thing I can do is to take my medicine faithfully.’ [Pt 38]	1. There is a known cure for diabetes^a^ 2. Diabetes can be reversed^a^ 3. Nothing can make my diabetes go away	**2.74 (1.28) (40.5% agreed) 2.10 (1.11) (74.7% agreed) 3.35 (1.36) (21.2% agreed)**	−0.25** −0.60** 0.46**
	Diabetes goes away if you exercise and lose weight	‘I know some people that were a diabetic, and they got a good, healthy, balanced diet and went to the gym, and they’re no longer on medicine or anything anymore. I’m believing that it’ll go away for me one day’ [Pt 25] ‘So if you, in other words, if you exercised and lost weight, there's a possibility your diabetes would go away’ [Pt 38]	4. My diabetes could go away if I exercise, lose weight and eat healthy ^a^	**2.24 (1.09) (68.2% agreed)**	−0.60**
Consequences: IP6 My diabetes is a serious condition IP7 My diabetes has major consequences on my life IP8^a^ My diabetes does not have much effect on my life IP9 My diabetes strongly affects the way others see me IP10 My diabetes has serious financial consequences IP11 My diabetes causes difficulties for those who are close to me	Relationship consequences	‘You don't be feeling it all the time like you used to … With my family and my woman. You know, like they think you’re a diabetic because you don't have no control over your life. And they like dictate you … you be in the restaurant ordering. They say, you can't have that … it changed the other person. They don't feel the same about me no more. They ain't paying attention to me no more.’ [Pt 06] ‘Well, for me, like he said, like the sexual drive, you know, it was a thing where just, you know, don't touch me.’ [Pt 18]	1. My diabetes reduces the control I have over my life 2. My diabetes has harmed my relationship with others close to me	**2.95 (1.24) (38.2% agreed) 3.55 (1.19) (19.6% agreed)**	0.61** 0.72**
	Friends/family relationship consequences	‘Or … you go out to eat with the girls, and they like … you shouldn't be drinking soda. You should be drinking water … you shouldn't even be thinking about dessert, or drinking … You know, yeah, I know … Thank you for being concerned, but I have this.’ [Pt 04] ^‘^And diabetes changes your family, because once they know you got it, they like always on me. Like if we go to a family event, I can't have anything. I can't even enjoy myself. … And like my young daughter, she going to have a baby … Then she went on to, oh, when my baby get here I don't know if I’m going to let you watch him.’ [Pt 06]	3. My diabetes has caused difficulties in my relationships with family and friends 4. My diabetes has caused my family and friends to be less close	**3.48 (1.21) (21.7% agreed) 3.61 (1.14) (15.3% agreed)**	0.75** 0.71**
	Lifestyle changes	‘I don't have the attention span to stay awake a long time after being at work all day. I just want to go home … Yeah. I’m very much, you know, social but a loner … I just don't have the mental capacity to do all that anymore’ [Pt 18]	5. My diabetes reduces my participation in social activities within the community	**3.19 (1.35) (33.5% agreed)**	0.66**
	Experiences with Food	‘I used to enjoy food, and I don't have that luxury anymore. That's gone.’ [Pt 03] ‘Well, like food is your enemy now’ [Pt 06]	6. My diabetes takes away the ability to enjoy food in my daily life	**2.74 (1.32) (50.0% agreed)**	0.66**
	Change of identity as an employee	‘Well, diabetes, it messed up my job, because I used to be a truck driver, and once I got diabetes, they’d want to take me off the road because I didn't have control over it … so I had to change jobs because of diabetes.’ [Pt 06]	7. Having diabetes has kept me away from the type of job I want to have	**3.35 (1.26) (31.8% agreed)**	0.71**
	Sociocultural consequences	‘The food that you like, so the things that you were brought up on. So all of your cultural things are out the window. You know, they’re like the forbidden … ’ [Pt 03]	8. My diabetes has taken away my ability to enjoy the food I grew up eating	**2.48 (1.29) (59.4% agreed)**	0.62**
Personal control: IP12 There is a lot which I can do to control my symptoms IP13 What I do can determine whether my diabetes gets better or worse IP14 The course of my diabetes depends on me IP15^a^ Nothing I do will affect my diabetes IP16 I have the power to influence my diabetes IP17^a^ My actions will have no effect on the outcome of my diabetes	Internal control	‘my grandmother told me, if you do not self-educate yourself, I feel sorry for you. And she told me if you let inequality of the races stop you from being who you are as a strong, black woman, she said, more better for you … she told me you cannot let that stop you from being you and taking care of yourself … ’ [Pt 11]	1. My diabetes is a big part of who I am 2. Having diabetes has made me feel less like a strong, black, person^a^	**2.81 (1.32) (42.3% agreed) 3.49 (1.27) (23.5% agreed)**	0.44** −0.04
		‘I can't think negative about it … So what I do is I try to find ways of survival … in my own mind, if I focus on the negative that it does to me, then I feel like I’m defeated, that I’m allowing the disease to take control of me … In this life, it's very hard, let alone having diabetes, and let alone being black and female in this country. So I can't afford to have something working against me, so I try to find the positives, and I try to do what I can … I’ve had to accept the things that I can change, and I have to have the courage to go through the things that I can't. I’m going to be all right. I ain't going to worry about it.’ [Pt 11]	3. It is important to not worry about my diabetes so as to protect my mental health.	**3.05 (1.39) (39.4% agreed)**	0.35**
	External control	‘Well, I believe, for me, that God plays a tremendous part in all of this. He gives me more knowledge. He takes me through this diabetes. God has a lot to do with it, but he carries me through it.’ [Pt 15] ‘If you got faith in God, yeah, that helps. It ease your mind … if you believe in God, you got faith in God … I would say, okay, good Lord, I have no control over this. All I can do is take my medicine. I’m putting this in your hands … So if I got faith in you, and I’m putting it in your hand, I’m not going to worry as much, because I know you got control of it. I got faith in you. You going to take care of me.’ [Pt 38] ‘It is so hard to have a whole bunch of black people together to strengthen one another … these type of groups that you’re having today, is necessary for the survival of people with diabetes, because networking and being together as a group is more powerful than trying to deal with it as one’ [Pt 11].	4. Faith in God helps control my diabetes 5. God helps me not to worry about my diabetes 6. My friends and family encourage me to manage my diabetes	**2.25 (1.18) (63.5% agreed) 2.19 (1.17) (67.6% agreed) 1.87 (0.89) (86.5% agreed)**	0.56** 0.54** 0.51**
Treatment control: IP19^a^ There is very little that can be done to improve my diabetes IP20 My treatment will be effective in curing my diabetes IP21 The negative effects of my diabetes can be prevented (avoided) by my treatment IP22 My treatment can control my diabetes IP23^a^ There is nothing which can help my condition	Importance of diabetes medication	‘The pills can help you maintain diabetes … ’ [Pt 03] ‘Well, we need them (medications) to survive … .’ [Pt 18] ‘Why would you stop taking something that you know is going to help you? Why would you not take something that you know that's going to help you? And I'm speaking about this, because I take mine religiously. The only reason I will not take it, if I simply forget., then, you know, they say, if you forget to take your medications just make sure you catch it the next go around … take your medication, you know.’ [Pt 34]	Medications can help me with my diabetes Medications can help me survive with my diabetes	**1.73 (0.89) (88.2% agreed)** **1.89 (1.00) (83.5% agreed)**	0.55** 0.55**
Illness coherence: IP24^a^ The symptoms of my condition are puzzling to me IP25^a^ My diabetes is a mystery to me IP26^a^ I don’t understand my diabetes IP27^a^ My diabetes doesn’t make any sense to me IP28 I have a clear picture or understanding of my condition	Understanding diabetes diagnosis with comparison of self to family member	‘My brother and sister ain't got it. And, man, how did I end up with it, and they didn't?’ [Pt 29] ‘ … that's something that the doctor should have been explaining to you why you can get diabetes, and you sit there in the same household, eating the same food … my husband, he eat candy like it's going out of style, you know, and eat everything in sight, and he don't have diabetes. I eat less than him, and I eat more fruits daily, vegetables, a little bit more of it … than he do, and … he just eats crazy stuff. And ain't nothing wrong with that man, like I say, that's not explained to us.’ [Pt 14]	How I got diabetes is a mystery to me^a^ I understand how I got diabetes	**2.80 (1.42) (41.2% agreed) 2.49 (1.22) (54.1% agreed)**	-0.61** 0.59**
Emotional representations: IP33 I get depressed when I think about my diabetes IP34 When I think about my diabetes I get upset IP35 My diabetes makes me feel angry IP36* My diabetes does not worry me IP37 Having this diabetes makes me feel anxious IP38 My diabetes makes me feel afraid	Fear of diabetes diagnosis and diabetes complications	‘My daughter expect to get it now. You know, she's pregnant now, but she always asking me about my diabetes … she's so scared of getting it … I think diabetes … scare you at first … like if you see somebody with their limbs off, they ain't got no foot. It was usually diabetes that did that … or I can't see now, I'm blind, because I had diabetes. I don't want to be like that.’ [Pt 06] ‘Right now, my father … to look at his legs … these people are talking about cutting … My brother, now they cutting on his feet … So I'm kind of wondering … I'm going to be in that situation one day … looking at my dad right now, getting ready to look like he's going to lose his legs. … it scared the shit out of me … it scared me to death.’ [Man 2]	I am worried about my children/ grandchildren getting diabetes The experiences of my family and friends has led me to fear diabetes complications I am scared of having complications from my diabetes	**2.52 (1.24) (61.8% agreed)** **2.85 (1.34) (43.5% agreed)** **2.36 (1.25) (63.5% agreed)**	0.56** 0.62** 0.66**
	Fear and doubt of the future	‘Me, I can't see what the future going to bring. All I can do is live right now, try to take care of this and make it to the future. Once I make it there, then I can keep going on, simple as that … I can't see it. I take it one day at a time.’ [Pt 38] ‘I want to be happy and then, my future … I want to be able to see my grandkids get older and graduate and stuff like that. That is my major … as long as I can talk to them and go see them and they going to see their papa.’ [Pt 4] ‘Well, I took it serious. When I found out I had diabetes … I was like I don't want to pass away. Because my mom passed away, I wanted to live as long as she did live at 83. I want to be her age. And … she was diabetic, that's why it made me serious.’ [Pt 41]	Having diabetes makes me worry about my future I am worried my diabetes will stop me from seeing my children and grandchildren grow up I am concerned about dying from my diabetes	**2.64 (1.34) (50.6% agreed)** **3.19 (1.28) (35.3% agreed)** **2.71 (1.30) (48.8% agreed)**	0.69** 0.65** 0.70**
	Anger and frustration with diabetes	‘When I got it, it was hard for me to accept it. I was angry … because I couldn't face the fact how come I have to be a diabetic, and I got to change all the things that I'm used to doing and should rearrange my whole life? And I was upset.’ [Pt 20] MAN 2: I'm about ready to give up … I am so sick and tired of … my sugar up that high … I don't eat too much sugar or eat no candy. I don't eat no sodas and stuff … and my sugar is steady going up on me. I'm trying to do what they tell me to do … but I can't do it	It is hard for me to accept that I have diabetes It makes me mad that I have to change my life because of diabetes I am frustrated while having diabetes	**3.05 (1.34) (38.2% agreed)** **2.77 (1.22) (47.1% agreed)** **2.57 (1.21) (56.4% agreed)**	0.70** 0.74** 0.72**
	Emotional dysregulation and depression	‘Sometimes your mood go up and down. Sometimes you have a good day, you know, nothing bother you, like sometimes you might have pain or burning in your feet. Some days you might not have that, you know. Seems like you be more angry’ [Pt 03]. ‘My doctor tells me, you know, [0025], you're kind of going into depression a little bit, because I stay in the bed all the time, because the neuropathy hurts so bad’ [Pt 25].	I am depressed because I have diabetes My diabetes controls my life I am upset I have diabetes	**3.01 (1.28) (35.8% agreed)** **2.99 (1.41) (32.3% agreed) 2.64 (1.40) (45.3% agreed)**	0.80** 0.68** 0.64**
	Perceived control and acceptance	‘I'm not going to let diabetes control me. My mom had diabetes. My dad had diabetes. My brother had diabetes. But I refuse to get myself depressed and upset because I have diabetes … I'm moving along with my life … I Live and let live. I'm not going to worry about it … you let the Lord handle it. He going to call you home when it's your time.’ [Pt 02] ‘I might have diabetes, but diabetes don't have me. I live my life and do what I got to do. Don't let it get you down, because, you'll be depressed. Keep doing what you doing. Eat right, exercise, and live a long life. Some people live to be 100 with diabetes. Some live longer than that. So I learned to say I got diabetes … but it don't have me. I'm not going to claim it, because I'm going to keep on living.' [Pt 20]	I refuse to be depressed because I have diabetes^a^	**2.19 (1.14) (69.4% agreed)**	**-0.09**
Additional Sociocultural Influences Domain	Role of Race, Racism, and Providers	‘They need to say the whole story, because you only getting half of the story when you get diagnosed. You know, if you don't attack it like you're supposed to, then the consequences, I always say I wish I would have listened to them, because this disease is troublesome. I'm always got to take my pills. I always got to do this before I eat. I can't have this, I can't have that. I didn't know that before.’ [Pt 03]. ‘(Racism) I think it influenced our lack of education about diabetes. So we don't have the same tools that we could use as preventative measures as non-black people would have.’ [Pt 03]	Being Black decreases my chances of knowing about diabetes control Being Black reduces my chances of getting information about diabetes Being Black makes me more likely to get diabetes	**3.28 (1.26) (28.8% agreed)** **3.24 (1.28) (30.6% agreed)** **2.91 (1.28) (42.3% agreed)**	0.75** 0.77** 0.61**
	Role of race and personal control of diabetes	‘This country is built on racism and discrimination. You have to be an advocate for yourself if you want to survive out here. Because they (other people-whites) have never had to suffer. They have never had to work, and they've always had choice, which is called white privilege. You see, we don't know what white privilege is … ’ [Pt 11]	As a Black person, I have to advocate for myself if I want to survive with diabetes	**2.42 (1.15) (61.8% agreed)**	0.37**
	The Role of the African American Community	**‘**Some people in the community there, and then some people want to be close-mouthed. They don't want to let people know that it's (diabetes) here. It's not nothing like you can put a Band-Aid one … It's a process of what you have, to learn how to control.’ [Pt 04] **‘**See, that's the kind of family I grew up in. … and they didn't even acknowledge, well, they didn't discuss diabetes or anything like this. There wasn't that many people with diabetes so, I mean, I didn't learn anything.’ [Pt 36]	Diabetes is a disease not discussed within the Black community My friends and family discuss diabetes ^a^ My friends and family discourage me from being open about my diabetes My friends and family help me learn about my diabetes ^a^	**2.89 (1.27) (41.1% agreed)** **2.51 (1.13) (56.4% agreed)** **3.46 (1.25) (21.1% agreed)** **2.39 (1.12) (64.1% agreed)**	0.56** -0.09 0.48** -0.19*
	Race and poverty	**‘**Yeah (a person’s race influence how people get diabetes), well, when you're borderline, you know, they just watch it. And I'm sure that if it was somebody else, they would show them the diet that they need to be on and the walking, … , I'm just thinking that that's how it goes for disadvantaged and anybody else.’ [Pt 04]	Being poor contributed to my getting diabetes	**3.54 (1.23) (22.4% agreed)**	0.68**

^b^ Bolded IPQ-R items were adapted in the study. Items not bolded were retained in the survey.

^a^ Old and New items reverse coded for scoring purposes.

*Significant at 0.05 level (2-tailed).

**Significant at 0.01 level (2-tailed).

### Quantitative results

Mean scores on 5-point Likert scale items ranged from 1.73 to 3.54 (where 1 = Strongly agree, 2 = Agree, 3 = Neither agree nor disagree, 4 =Disagree, and 5 = Strongly Disagree). Mean scores on the new survey items and the percentage of respondents agreeing with the culturally adapted IPQ-R survey items indicate the congruence between themes from the qualitative data and the subsequent quantitative data. Percentage of participants agreeing to the items ranged from 15.5% to 86.4% across all culturally adapted IPQ-R items. Item-total correlations for each culturally adapted IPQ-R item indicates the internal consistency of the newly developed items within their respective domains. All item-total correlations were statistically significant, except three reverse coded items. The significant Pearson's correlation coefficient values ranged from −0.61 to 0.80, with most items having moderate correlations. As expected, the negative correlations were for items worded to be in the opposite direction as compared to other items within the domain.

## Discussion

In this mixed methods study, culturally adapted survey items were created to assess the illness perceptions of African Americans about diabetes based on the self-regulatory model and the IPQ-R questionnaire. The qualitative phase resulted in quotes and themes that were used to create items for the quantitative survey. The influence of sociocultural factors such as the role of religion, family, food, experiences of discrimination and racism informed both the inductive and deductive themes, leading to the cultural adaptation of the questionnaire. Hence, integration of both qualitative and quantitative phases resulted in the structuring of culturally adapted IPQ-R items within the original IPQ-R questionnaire domains and the creation of a new sociocultural domain. Integration also provided data regarding the congruence between the qualitative and quantitative results, further aligning the results in both phases.

### Qualitative

The initial qualitative phase was essential to explore illness perceptions that may be unique to African Americans. Themes of various illness perceptions such as diabetes timeline perceptions, food and lifestyle consequences, understanding the disease with respect to family members’ experiences, fear of future complications, and feelings of anger and frustration, were all informed by lived experiences and sociocultural factors unique to African Americans. It was important to explore these illness perceptions because of their relationship to other diabetes outcomes including glycemic control, self-efficacy, and diabetes distress (Martinez, Lockhart, Davies, Lindsay, & Dempster, 2018) as well as self-management behaviors such as medication adherence, diet, and exercise (Kucukarslan, 2012). By addressing these unique illness perceptions among African Americans, it allows for the creation of culturally adapted self-management behavior interventions.

Research indicates that food and lifestyle changes have been important factors in the personal and socio-cultural lives of African Americans, especially in relation to diabetes (James, 2004). The participants in this study discussed the changes they had to make because of diabetes including missing out on enjoying culturally relevant food in family/social gatherings. Family support was an important theme in participants perceived personal control of diabetes, but some participants also expressed negative consequences on their social relationships due to having diabetes (Shiyanbola et al., 2018a, 2018b). Research has showed that diet and lifestyle restrictions imposed by family members are commonly seen among African Americans with diabetes (James, 2004). The link between food and family are sociocultural factors that are not accounted for in the original IPQ-R questionnaire.

The role of race and racism in patient-provider relationships, perceived personal control of illness, the influence of the African American community in diabetes management, and the perceived influence of poverty in diabetes, informed the new illness perception domain, i.e. sociocultural influences on diabetes. Participants described a lack of knowledge of diabetes stemming from discrimination from providers who may have withheld relevant education/information about diabetes. A prior study described coping behaviors among African American women with diabetes in response to discriminatory health care access (Murry et al., 2003). These behaviors included an exaggerated need to be in control and be self-reliant at the expense of recommended self-management behaviors (Murry et al., 2003). Participants in our study expressed similar perceptions about needing higher personal control and being an advocate for their own health because of racism in the health care system. Participants also expressed stigma from the African American community and distress regarding openly discussing diabetes. These findings are similar to previous research indicating the presence of overall stigma, blame, and self-stigma among African Americans with diabetes, especially those who were obese (Piatt, 2019). These cultural factors influencing illness perceptions are accounted for in the adapted IPQ-R.

### Quantitative

The average item scores and significant item-total correlations were important indicators of the initial validity and reliability of the culturally adapted survey items in this population. Most items had average scores ranging from 2.0 to 3.5, (with the extreme means being 1.73–3.61) indicating that participants in general found the items relevant to their illness perceptions. Most items did not have extreme average scores, an indicator of item quality when the goal of the assessment is normative. Generally, items with extreme average scores (and thus a lack of variance on a Likert scale) would not provide useful comparative information about respondents. Only three culturally adapted items had somewhat extreme average scores. These survey items evaluated perceptions about necessity of medications under the treatment control domain (two items), and the role of family/friends as an external influence in their personal control of diabetes (one item). Interestingly, research indicates that African Americans commonly have high concerns about their diabetes medications, which is expected to be negatively associated with necessity of medications (Aikens & Piette, 2009; Shiyanbola et al., 2018a). Thus, the extreme scores are consistent with expectations, and it was considered important to retain those two items due to their descriptive characterization of the population and to further explore this perception via studying beliefs in medicines specifically. Also, research shows that social support has positive effects on diabetes self-management behaviors in African Americans. However, the relationship between social support and perceived personal control on diabetes management has not been studied. Hence, the third item with extreme scores may also need to be retained. Finally, with regards to the survey domain item-total correlations, the significant values indicate that the culturally adapted items have good reliability. Interestingly, the only items that had non-significant correlation values were items that needed to be reverse coded for total scoring purposes. This could possibly be due to acquiescence bias or misinterpretations of the directionality of the item.

### Integration

Integration of qualitative and quantitative data occurred at two points, first when using the building approach to create new culturally adapted items and then when using the merging approach to report the results through the joint display. The cultural adaptation involved the rewording of existing items, as well as the creation of new items. Creating culturally adapted items based on in-depth qualitative data resulted in increased content validity of the items. Firstly, matching the themes to the ESRM theoretical domains led to a streamlined approach to the development and structuring of survey items. However, research indicates that the factor structure of the IPQ-R evaluated in a general population may not apply to an African American population (Abubakari et al., 2012; Ward et al., 2013). Therefore, it is possible that the factor structure of the fully developed culturally adapted IPQ-R is not the same as the original IPQ-R, despite matching the themes with domains. Future research that evaluates the construct validity of the culturally adapted IPQ-R questionnaire through exploratory factor analysis is needed. Another important advantage of integration using the building approach was the use of actual participant quotes to form the survey items. This resulted in culturally adapted items that used preferences of the population, as well as cultural nuances that reduced item misunderstanding and subsequent measurement errors. Finally, using the merging approach to integration during reporting highlighted the congruence between the qualitative and quantitative results, thereby making our previous qualitative findings more reliable.

### Implications

The development of a culturally adapted IPQ-R improves the assessment of African Americans’ illness perceptions as it accounts for the influence of sociocultural factors including the role of family, religion, food, discrimination and mistrust in the healthcare system etc. The initial qualitative focus groups allowed for the understanding of beliefs and perceptions of African Americans regarding diabetes, which influenced the cultural adaptation of the IPQ-R.

The development of this adapted questionnaire is important for healthcare providers to understand how African Americans’ cultural contexts and beliefs might influence their perceptions about diabetes and shape their self-management behaviors including medication use and adherence, therefore, allowing for tailored interventions, improved clinical outcomes, and reductions in diabetes health disparities.

## Limitations

There were some limitations to the study. Firstly, the length of the survey could have caused respondent burden and subsequent measurement errors. The increase in length was mainly due to the additional items under the sociocultural domain. Also, in some cases, one survey item led to the creation of multiple items after the adaptation. For example, the item ‘This diabetes will pass quickly’ was adapted into ‘There is a known cure for diabetes’ and ‘Diabetes can be reversed’ to better capture the actual underlying perceptions of how diabetes will ‘pass quickly.’ It was also important to retain the original IPQ-R items that didn't need to be culturally adapted to accurately evaluate all aspects of illness perceptions among the study population. To avoid increasing respondent burden further, IPQ-R items that were adapted and no longer relevant to the study population were not included in the survey. However, this meant that no item-level comparisons could be made between old and adapted items. Secondly, this study population was limited to a middle-aged African American population from a Midwestern state. Although we reached saturation in our qualitative focus groups, the quantitative results could have differed if the items were tested in a different sample. Finally, this study focuses on integration using initial item-level quantitative results. More quantitative analysis and psychometric testing are required before final questionnaire development.

Despite these limitations, this study uniquely contributes to the fields of instrument development, mixed methods, socio-behavioral research, and health equity. Although many tools are routinely adapted for different populations, the use of rigorous mixed methods in the cultural adaptation of an instrument is limited. Many studies use qualitative data to develop instruments, but fail to integrate their findings correctly, do not systematically use a building approach to form items, and rarely merge findings when reporting results. Moreover, this is the first mixed methods study exploring African Americans with diabetes perceptions to create culturally adapted items based on the IPQ-R. Developing a tool specifically for African Americans with diabetes will lead to an accurate assessment of illness perceptions and help in the creation of tailored interventions to improve outcomes such as medication adherence, patient-provider relationships and communication, diabetes self-management behaviors, and overall diabetes care. Improved interventions for African Americans with diabetes may lead to a reduction in diabetes disparities.

## Conclusion

The study used an exploratory sequential mixed methods design to develop and adapt survey items from an existing illness perception questionnaire, based on qualitative themes assessing perceptions of diabetes among African Americans. This paper also describes the process of integrating qualitative and quantitative data in the methods and reporting stages. The newly developed items will be used to finalize the culturally adapted IPQ-R after psychometric testing and questionnaire refinement. Future use of the questionnaire includes improving health outcomes in African Americans via culturally tailored interventions to change negative illness perceptions, using the culturally adapted IPQ-R for other illnesses, and creating shorter versions that can be readily integrated into clinical practice to assess illness perceptions of African Americans.
